# The changing face of immune tolerance induction in haemophilia A with the advent of emicizumab

**DOI:** 10.1111/hae.13762

**Published:** 2019-04-29

**Authors:** Manuel Carcao, Carmen Escuriola‐Ettingshausen, Elena Santagostino, Johannes Oldenburg, Ri Liesner, Beatrice Nolan, Angelika Bátorová, Saturnino Haya, Guy Young, Future of Immunotolerance Treatment Group

**Affiliations:** ^1^ Division of Haematology/Oncology, Department of Paediatrics and Child Health Evaluative Sciences, Research Institute, The Hospital for Sick Children University of Toronto Toronto Ontario Canada; ^2^ Haemophilia Centre Rhine Main Möerfelden‐Walldorf Germany; ^3^ Maggiore Hospital Policlinico Angelo Bianchi Bonomi Hemophilia and Thrombosis Center Milan Italy; ^4^ Institute of Experimental Haematology and Transfusion Medicine University of Bonn Bonn Germany; ^5^ Haemophilia Centre, Great Ormond Street Hospital for Children NHS Trust Haemophilia Centre London UK; ^6^ Our Lady's Children's Hospital Crumlin Dublin Ireland; ^7^ Department of Haematology and Transfusion Medicine and National Haemophilia Centre University Hospital, Comenius University Bratislava Slovakia; ^8^ Unit for Congenital Bleeding Disorders Hospital Universitario y Politécnico La Fe Valencia Spain; ^9^ Children's Hospital Los Angeles University of Southern California Keck School of Medicine Los Angeles California

**Keywords:** bypassing agents, emicizumab, factor VIII, haemophilia A, immune tolerance induction, inhibitor

## Abstract

**Introduction:**

As a result of the new treatment paradigm that the haemophilia community will face with the availability of novel (non‐factor) therapies, an updated consensus on ITI recommendations and inhibitor management strategies is needed.

**Aim:**

The Future of Immunotolerance Treatment (FIT) group was established to contemplate, determine and recommend the best management options for patients with haemophilia A and inhibitors.

**Discussion and Conclusions:**

Despite the considerable success of emicizumab in the management of inhibitor patients, the FIT group still sees the importance of eradicating inhibitors. However, the availability of emicizumab and other non‐factor therapies in the future might impact greatly on how ITI is undertaken. Theoretically, concomitant use of emicizumab and FVIII might allow emicizumab to effectively prevent bleeding with lower dose ITI regimens. This might allow for the greater adoption of low‐dose/low‐frequency FVIII ITI regimens, which may result in a reduced need for central venous access devices while still maintaining a reasonable likelihood of ITI success. The FIT group proposes a new management algorithm for current ITI (without emicizumab) and a hypothetical new approach with the availability of emicizumab. As there are no published data regarding the concomitant use of emicizumab and FVIII for ITI, the FIT Expert group encourages the undertaking of properly conducted prospective studies to explore these approaches further.

## 1 INTRODUCTION

The development of neutralizing antibodies (inhibitors) against factor VIII (FVIII) occurs in 25%‐40% of patients with severe haemophilia A.^1^, ^2^, ^3^, ^4^, ^5^ Persons with haemophilia A who develop high‐titre inhibitors (HTI) become resistant to FVIII replacement therapy. This is associated with increased risk for bleeding and resultant morbidity (severe arthropathy and disability) and increased mortality.^6^, ^7^, ^8^ Studies have shown that haemophilia‐related long‐term morbidity and mortality as well as long‐term costs are diminished if inhibitors are eradicated.^9^

The only proven strategy for achieving inhibitor eradication is immune tolerance induction (ITI), involving repeated administration of FVIII concentrates.^10^, ^11^, ^12^ In 2007, DiMichele et al^12^ developed a management algorithm and published consensus recommendations for ITI in persons with haemophilia A and inhibitors. Since that time, however, the treatment of haemophilia A has evolved and a number of molecules that potentially can be used in the setting of patients with inhibitors have been developed, or are in various phases of development.^13^, ^14^, ^15^, ^16^ With the arrival of these new molecules, the treatment environment is changing, and there are many unanswered questions about the future of inhibitor management. To provide answers to these and other questions, a group of nine experienced haemophilia treaters came together to discuss the Future of Immunotolerance Treatment (FIT) and to provide some orientation to the haemophilia community in this changing environment.

### 1.1 The Fit group

The FIT group was formed in 2017 by Grifols to gain insight into how inhibitor management might change with the advent of new haemophilia therapies. Potential members were identified on the basis of their expertise in inhibitor management, their history of publishing on the subject and the fact that they represented large haemophilia centres. Identified members were invited to participate by Grifols. No individual invited to participate declined the invitation. The group was limited to nine members as anything larger would be unmanageable. Three meetings were conducted between November 2017 and July 2018. Based on the transcripts of these meetings, a medical writer developed an initial draft manuscript. After that, the nine members took over the development of the paper with no further involvement from Grifols personnel or hired medical writers. As high‐level evidence regarding the addition of emicizumab or other non‐factor therapies to inhibitor management is currently lacking, the recommendations offered by the FIT group reflect consensus opinions of the members.

This report collects the group's current views and recommendations for the management of inhibitors without (Part A) and with (Part B) the addition of non‐replacement therapies, respectively.

## 2 PART A: FIT GROUP APPRAISAL OF CURRENT ITI

### 2.1 ITI objectives

The objectives of ITI are to eradicate the inhibitor and thus avoid the complications associated with a lifelong inhibitor.

### 2.2 Which patients are candidates for ITI?

**Figure 1:**
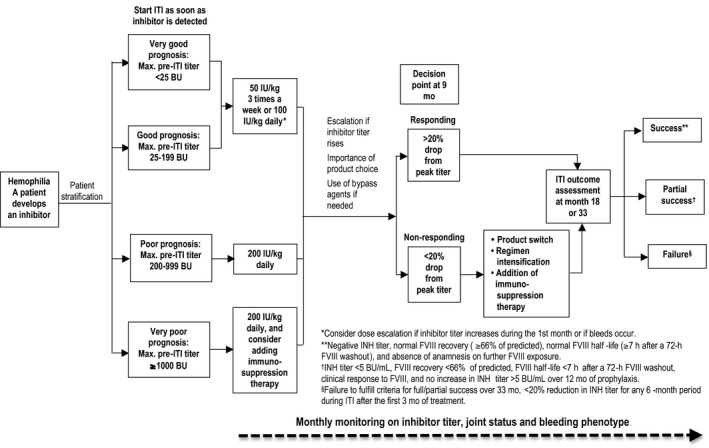
FIT proposed management algorithm for current ITI. *Consider dose escalation if inhibitor titer increases during the 1st month or if bleeds occur. **Negative INH titer, normal FVIII recovery (≥66% of predicted), normal FVIII half‐life (≥7 h after a 72‐h FVIII washout), and absence of anamnesis on further FVIII exposure. ^†^INH titer <5 BU/mL, FVIII recovery <66% of predicted, FVIII half‐life <7 h after a 72‐h FVIII washout, clinical response to FVIII, and no increase in INH titer >5 BU/mL over 12 mo of prophylaxis. ^§^Failure to fulfill criteria for full/partial success over 33 mo, <20% reduction in INH titer for any 6‐mo period during ITI after the first 3 mo of treatment

The many problems associated with inhibitors are compounded by the very young age of patients (in most cases) who develop inhibitors, which typically occurs during the first 20‐40 exposure days (EDs) to FVIII replacement.^5^, ^17^ Eradication of inhibitors through ITI has been considered essential in young children developing HTI. However, even in older children and adults with severe haemophilia, ITI also has been considered appropriate in several settings: (a) adults with recent inhibitor development due to previous infrequent FVIII exposure, (b) young and older patients with long‐standing inhibitors who never attempted ITI and (c) patients with a history of failed ITI for whom rescue ITI might still be effective. The FIT group's proposed management algorithm for current ITI is shown in Figure 1.

### 2.3 ITI success rates

The success rate of ITI has been demonstrated to range between 60% and 80% in patients with severe haemophilia A,^11^, ^18^, ^19^ although when analysed on an intent‐to‐treat basis, the success rate appears to be lower.^11^ When a patient is not responding to a first ITI regimen, a change in regimen (eg, dose escalation) or a change in FVIII product and/or the addition of an immunosuppressant (eg, rituximab) has been considered appropriate. Traditionally, haemophilia treaters, recognizing the importance of eradicating the inhibitor, have tried repeated attempts at ITI when inhibitor eradication is not initially achieved.

### 2.4 Predictors of ITI success

Much of the awareness of predictors of ITI success arose from several large registries of ITI: an International ITI registry,^20^ a German registry^21^ and a North American registry.^22^ Based on the findings from these registries, DiMichele et al^12^ recommended in 2007 that patients be classified into low‐ and high‐risk categories (for ITI success) based on historical pre‐ITI peak inhibitor titre, inhibitor titre immediately prior to starting ITI and time since inhibitor diagnosis to start of ITI. The FIT group (now 12 years later) concurs with DiMichele et al that historical pre‐ITI peak inhibitor titre and peak inhibitor titre while on ITI are independent predictors of ITI success/failure, but we no longer believe that the inhibitor titre immediately prior to starting ITI on its own is an independent predictor of ITI success/failure given some recent publications and unpublished experiences of FIT group members of starting ITI in patients with HTI without waiting for titres to fall. Other putative prognostic factors, also mentioned for risk stratification by several authors, we regard (much like DiMichele et al did) as being less important.^8^, ^23^

### 2.5 When to start ITI

An increasing number of clinicians, including those in the FIT group, have recently elected to start ITI immediately (provided inhibitor titres are >5 BU) regardless of how high the inhibitor titre is. It should be noted that ITI is sometimes commenced in patients with low titre inhibitors (LTI; <5 BU). FIT group members believe, however, that there is good evidence to suggest that many of these LTI will resolve without the need for ITI and as such we advocate not starting ITI for LTIs except in certain circumstances (eg, when patients are experiencing excessive bleeding). There is even some evidence of spontaneous disappearance of inhibitors when inhibitor titres are between 5 and <10 BU.^24^

Our recommendation of starting ITI immediately is in contrast to past practice, where most clinicians would wait until the inhibitor titre had dropped to a value of <10 BU.^11^, ^12^ This trend of starting ITI as soon as possible after inhibitor development has been driven in part by a desire to avoid bleeds in patients with high pre‐ITI titres^25^ and in the hope of suppressing the maturation of the anti‐FVIII immune response with the production of long‐lived plasma cells.^26^

### 2.6 ITI patient risk group stratification

**Figure 2:**
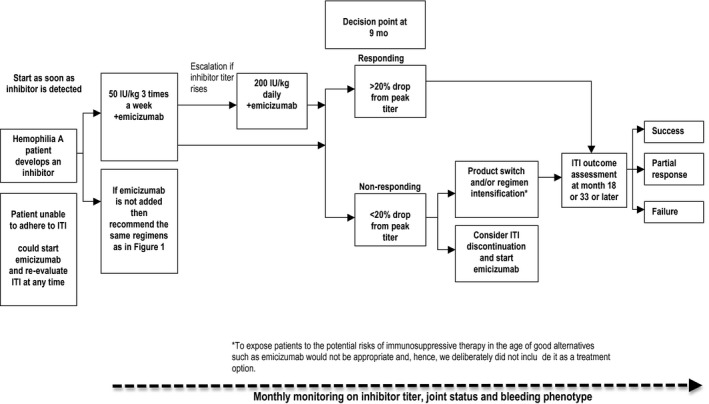
Hypothetical new approach for the management of inhibitor patients in the era of non‐factor therapies. *To expose patients to the potential risks of immunosuppressive therapy in the age of good alternatives such as emicizumab would not be appropriate and, hence, we deliberately did not include it as a treatment

DiMichele et al^12^ classified patients into two groups: ‘good risk’ (patients with historical peak inhibitor titre <200 BU, an immediate pre‐ITI inhibitor titre <10 BU and time from inhibitor development to start of ITI of <5 years) and ‘poor risk’ (patients with historical peak inhibitor titre >200 BU, immediate pre‐ITI titre >10 BU, or >5 years since inhibitor diagnosis). The FIT Group concurs with DiMichele et al to stratify patients but recommends stratifying patients into four groups instead of two, based only on historical pre‐ITI peak titre (Figure 1): <25 BU (very good prognosis), 25‐<200 BU (good prognosis), 200‐<1000 BU/mL (poor prognosis) and ≥1000 BU (very poor prognosis). The 25 BU cut‐off was chosen as the International (I) ITI study^11^ showed that the likelihood of ITI success was statistically much higher if patients had a peak inhibitor titre pre‐ITI of <23 vs ≥23 BU. Given the awkward nature of 23 BU, we chose 25 BU. The choice of 200 BU came from DiMichele et al, who used this in the International (I) ITI study, while the choice of >1000 BU as constituting a very poor prognosis group is based on the personal experience of FIT Group members.^27^ These new prognostic groups translate to new initial ITI regimen selection recommendations (see Figures 1 and 2).

### 2.7 Product selection

It has been common practice to use the same FVIII concentrate for ITI to which the patient originally developed the inhibitor.^28^ However, the FIT group does not believe that there is any corroborating evidence to support or refute this practice. In fact, several reports suggest a high rate of ITI success when a plasma‐derived (pd) FVIII/VWF is used for first‐time ITI in patients who develop an inhibitor with recombinant (r) FVIII.^8^, ^27^, ^29^, ^30^ However, given the lack of randomized studies comparing the efficacy of different FVIII product types for first‐time ITI, the FIT group currently cannot recommend a specific FVIII product or product type for first‐time ITI patients.

### 2.8 Dose and regimen

There are two main approaches when starting patients on ITI: (a) begin ITI with a non‐tailored prespecified regimen regardless of the patient's initial prognostic status or (b) select the starting regimen that is tailored to the patient's prognostic status. The FIT group recommends the second approach and, as shown in Figure 1, defines different dose/regimen options**.** Low‐dose/low‐frequency (eg, 25‐50 U/kg 3 times/wk) or intermediate‐dose/high‐frequency (100 U/kg daily) regimens may be good options for patients with a very good or good prognosis, respectively. Low‐dose/low‐frequency regimens in such patients have shown long‐term effectiveness similar to that of high‐dose regimens.^11^ The shortcomings of low‐dose/low‐frequency regimens are that they are associated with more bleeds (in the I‐ITI study,^11^ the rate of bleeding in the low‐dose arm was 2‐fold higher than the rate of bleeding in the high‐dose arm: 0.62 vs 0.28 bleeds/mo) and take longer to achieve inhibitor tolerance (in the I‐ITI study 15.5 vs 10.6 months). These drawbacks may be balanced by such regimens being more convenient for patients, resulting in a lower need for central venous access devices (CVAD) and being substantially less costly than high‐dose/high‐frequency regimens, even if bypassing agent prophylaxis is given concomitantly with low‐dose/low‐frequency regimens.

Our group recommends that high‐dose/high‐frequency regimens such as 200 IU/kg/d as a single dose or split into twice/day dosing be used for poor and especially for very poor prognosis patients; it is in the latter group of patients that immunosuppression traditionally has been considered. This recommendation is based on the fact that there is experience with successful ITI when high‐dose regimens are used in patients with inhibitor titres >200 BU, while there are no reports of success when low‐dose ITI regimens are used in such patients.

### 2.9 ITI monitoring and decision points to adjust ITI

Being on ITI is a state of constant flux, with inhibitor titres rising or falling depending on the patient's response to ITI. Given this, it is important that inhibitor titre testing and monitoring including joint status and bleed phenotype be performed on a frequent (eg, monthly) basis, and dose/frequency escalation should be considered based on a substantial rise in inhibitor titres or the occurrence of significant bleeding. We concur with the approach taken by the UKHCDO group, which indicated that low‐risk patients who commence a low‐dose ITI regimen (50 U/kg on alternate days) should be escalated to 100 U/kg daily should their inhibitor titre rise to >40 BU and for those patients commencing on 100 U/kg daily that their regimen should be escalated to 200 U/kg daily if their inhibitor titres rise to >200 BU while on ITI.^31^

In addition to the ongoing monitoring of inhibitor titres and escalation of ITI as indicated above, there are certain key times when important decisions regarding an individual's ITI regimen should be made. It has been suggested previously, and the FIT group concurs, that 9 months after starting ITI is a key decision point, as it might allow a patient to be declared as failing ITI if they have not shown a sufficient decrease (minimum of 20% has been suggested) in inhibitor titre from start of ITI. It is at this point that major alterations to the management plan should be considered in patients declared to be failing; this might involve intensifying the ITI regimen (dose and/or frequency), changing the FVIII product being used for ITI, adding immunosuppression or some combination of these.

### 2.10 Prophylaxis with bypassing agents during ITI

Prophylaxis with standard bypassing agents (recombinant activated FVII [rFVIIa] and plasma‐derived activated prothrombin complex concentrate [FEIBA]) should be considered on a case‐by‐case basis according to the patient's bleeding pattern.^32^ ITI itself has been shown to reduce rates of bleeding particularly when the inhibitor titre is not very elevated.

### 2.11 Central venous access devices (CVADs)

The need for a CVAD generally depends on the ITI dosing regimen and on the patient's age. As CVADs are associated with well‐known complications (local and systemic infections, mechanical device failure and thrombosis), peripheral venous access generally should be used whenever possible.^33^ However, the experience of FIT group members is that it is almost impossible to undertake high‐dose/high‐frequency ITI regimens in young children without a CVAD.

### 2.12 Success definition

The FIT group agrees that the response to ITI should be defined generally according to the criteria of DiMichele et al—success, partial success and failure—as delineated in Figure 1.^12^ The only potential change to this concerns what half‐life should be used to declare inhibitor negativity. Some studies have suggested a half‐life of 7 hours to define normal half‐life, and some consideration exists to potentially extend this with extended half‐life FVIII. For now, given the absence of definitive data, the FIT group believes that 7 hours should be the criterion for a normal half‐life.

### 2.13 When to discontinue ITI

The maximum time limit for ITI has not been established. Some studies have suggested that 33 months might represent a maximal time to undertake ITI as after 33 months few additional patients are likely to achieve ITI success.^34^

## 3 PART B: FIT GROUP REFLECTIONS AND RECOMMENDATIONS FOR ITI IN THE ERA OF NON‐FACTOR THERAPIES

Newer, non‐factor therapies that can be used in patients with inhibitors have been developed and are likely to impact greatly on the practice of ITI in the future. Emicizumab, the first of these non‐factor therapies to be licensed for inhibitor patients, is a bispecific antibody that binds to both factor IXa and factor X and in doing so supports conformational changes that allows factor IXa to activate FX in the absence of FVIII.^35^ The efficacy of emicizumab in preventing bleeds has been demonstrated to be much higher than that of traditional bypassing agents.^13^ On the basis of this, emicizumab was approved in November 2017 by the FDA and in February 2018 by the EMA for prophylactic use in adults and children with haemophilia A and inhibitors to FVIII. It has recently been approved (October 2018 by the FDA and January 2019 by the EMA) for use in patients with haemophilia A without inhibitors. The convenience (subcutaneous infusions given once/week, once every 2 weeks or once every 4 weeks) of emicizumab stands in stark contrast to the gruelling treatment burden of ITI and bypassing agent prophylaxis. This together with the fact that emicizumab is efficacious in preventing bleeds in both patients with and without inhibitors has raised myriad questions (Table 1).

**Table 1:** Questions regarding ITI facing the haemophilia community with the advent of non‐factor therapies

Should patients with inhibitors still undergo one or multiple ITI attempts to eradicate their inhibitors?
Should emicizumab be given concurrently with ITI to prevent bleeds?
Given that now with emicizumab low‐dose ITI regimens may no longer be handicapped by higher bleeding rates in comparison with high‐dose ITI regimens (which are associated with much higher cost and burden) will ITI regimens change?
Will government and insurance payers support the cost of concomitant emicizumab with ITI?
Will there be any role for prophylaxis with traditional bypassing agents (rFVIIa and FEIBA)?
If patients undergo ITI (particularly patients receiving concomitant emicizumab) and achieve success, will they continue on emicizumab?
If patients remain on emicizumab post inhibitor eradication, must they continue on some regular exposure to FVIII to maintain tolerance to FVIII?

The FIT group's vision about a hypothetical new approach to the management of inhibitor patients in the era of non‐factor therapies is shown by the new algorithm in Figure 2. The FIT group's reflections considered only clinical implications of emicizumab in the management of inhibitor patients, but the group acknowledges that there also will be cost implications that should be considered on a country‐by‐country basis.

### 3.1 Tolerization is still the goal

With the availability of emicizumab, and, in the future, other non‐factor therapies, the first decision that a clinician will grapple with if a patient develops a HTI is whether ITI should still be attempted (with or without concomitant emicizumab/non‐factor therapy) or should we not bother with ITI and instead place the patient immediately on emicizumab alone.

There is no evidence and no expectation that emicizumab given alone will result in eradication of inhibitors although it is possible that some inhibitors will resolve without ITI (addressed earlier). However, for any inhibitor titre >10 BU, if patients/clinicians were to choose not to undertake ITI, such patients would (we believe) have an inhibitor for the rest of their lives.

Although emicizumab, and in the future other non‐factor therapies, may ultimately be shown to be excellent long‐term prophylactic agents for patients with haemophilia A with and without inhibitors, they do not appear (for now) to be able to prevent all bleeds. Consequently, bleeds are still likely to occur and will require additional episodic treatment: FVIII (if patient is inhibitor‐negative or has a LTI) or bypassing agents (if patient has a HTI). The recently reported experience of Mahlangu et al where FVIII was given to patients without inhibitors on emicizumab (215 events) without any thrombotic complications suggests that treating bleeds with FVIII in patients on emicizumab in whom the inhibitor has been eradicated is likely to be both safer and more convenient than treating bleeds with current bypassing agents (rFVIIa or FEIBA) in patients on emicizumab in whom the inhibitor persists.^36^ The same would apply to patients undergoing surgery. Therefore, recognizing the value of not having an inhibitor, the FIT group believes that all patients with inhibitors should still be offered at least one attempt at ITI. Ultimately, it is the decision of the patient/family, and given the considerable demands of ITI, emicizumab alone without an attempt at ITI is an alternative.

### 3.2 Concomitant use of emicizumab and ITI

Although the efficacy and safety of emicizumab given together with FVIII during ITI have not been evaluated in clinical studies, there are a growing number of case reports on such use. Lenting et al^35^ reviewing the similarities and differences between FVIII and emicizumab from biochemical and mechanistic perspectives concluded that there should be no reason why FVIII and emicizumab could not be used together, as they compete for the same substrates, FIXa and FX, and thus when used together will result in minimal additive haemostatic effects. The intermittent use of FVIII (in the setting of bleeds/surgeries) in patients on emicizumab (without inhibitors) published by Mahlangu et al^36^ and alluded to earlier confirmed that the combined use of FVIII with emicizumab is safe as predicted by Lenting et al.^35^

Theoretically, concomitant use of emicizumab and FVIII as part of ITI would allow emicizumab to prevent bleeding while FVIII would be used solely to induce tolerance. This could permit greater use of low‐dose/low‐frequency ITI regimens, thereby avoiding the need for CVADs while still maintaining the likelihood of successful ITI. As such, in our new algorithm (Figure 2), we have indicated that one option might be to start patients on low‐dose ITI with emicizumab regardless of their historical peak inhibitor titre. Patients could then escalate their ITI regimen should their response to low‐dose ITI be deemed insufficient as per the earlier discussion.

There are many ramifications of adding concomitant emicizumab to ITI. First, the overall cost of ITI could rise considerably, particularly if high‐dose ITI is chosen although a low‐dose ITI regimen plus emicizumab may be less costly than current high‐dose ITI regimens due to savings on FVIII use and potential savings from less use of traditional bypassing agents. Much of the cost implications will be determined by the cost of emicizumab on a per mg basis, by the unit cost of FVIII concentrates and of traditional bypassing agents. It will be important to capture these costs in future prospective studies of ITI incorporating emicizumab.

Second, if emicizumab is added to ITI, it will likely impact which bypassing agent will be used to manage episodic bleeds; it should be noted, however, that the frequency of bleeds in paediatric patients on emicizumab is quite low (ABR of 0.3).^14^ Lastly, there will be a need for special laboratory monitoring utilizing a bovine chromogenic FVIII assay to measure both FVIII inhibitor levels as well as FVIII levels when undertaking pharmacokinetic testing in patients on ITI.

### 3.3 Bleed management of patients on concomitant emicizumab and ITI

In clinical trials of emicizumab in inhibitor patients, several patients developed thrombotic microangiopathy or thrombotic events in the setting of receiving multiple high doses of FEIBA over a period of >24 hours.^37^ Given this, our group would recommend that rFVIIa be the preferred bypassing agent in the setting of emicizumab and that FEIBA should only be used in situations where rFVIIa is ineffective and with very close supervision. If emicizumab is incorporated into ITI, it should be understood that emicizumab takes 4‐5 weeks to establish a steady state level and become fully effective.^13^

### 3.4 What to do after completion of ITI?

If a patient on ITI and concomitant emicizumab is successful in inhibitor eradication, how is the patient to be managed following successful inhibitor eradication? Should the patient resume FVIII prophylaxis (generally receiving 2‐3 intravenous infusions per week) or continue weekly (or less frequent) subcutaneous emicizumab with or without ‘regular’ FVIII? Given the ease of emicizumab administration, we believe that many/most patients (if successful with ITI) might want to continue on emicizumab rather than stop emicizumab and return to frequent intravenous injections of FVIII. If patients remain on emicizumab, it is not known what amount of regular FVIII exposure (if any) might be needed to maintain tolerance and for how long such regular FVIII exposure would be required.

Given the absence of data on this, our group would propose that following achievement of tolerance that patients be maintained on a minimum of once‐weekly FVIII infusions for at least 6 months followed by a further 6 months of once every 2‐week dosing. During this time, testing for neutralizing (and where possible non‐neutralizing) anti‐FVIII antibodies should be done every 1‐2 months along with recovery testing every 2‐4 months. After this, regular exposure to FVIII could be discontinued with ongoing surveillance for anti‐FVIII antibody development with FVIII pharmacokinetic studies (every 3‐6 months). If enough clinicians adopt a uniform approach such as this (ideally as part of a prospective study), then the haemophilia community may come to better understand if regular FVIII exposure is needed to maintain tolerance.

Alternatively, if tolerization is not achieved, should additional ITI attempts be offered? With the availability of emicizumab and other non‐factor therapies, and recognizing the demanding and costly nature of ITI, we believe that clinicians, patients/families and/or payers will likely limit ITI to only one attempt. Therefore, every attempt should be made to ensure success with the first course of ITI.

## 4 CONCLUSIONS AND RECOMMENDATIONS

Since the first report of ITI in the 1970s, the haemophilia community has adopted ITI as standard of care when patients with severe haemophilia A develop HTIs. ITI has for the past almost 50 years remained relatively unchanged. Now, non‐factor therapies beginning with emicizumab raise many questions. There is a need to provide haemophilia treaters with guidance in this changing therapeutic environment. The FIT group's key conclusions are summarized in Table 2. The FIT group believes that inhibitor eradication remains an important goal and that ITI should be individualized according to a patient's predictors for ITI success. Concomitant use of emicizumab during ITI is a new alternative to prevent bleeds while eradicating inhibitors. Such an approach is likely to influence the choice of ITI regimen with a greater likelihood of lower dose/lower frequency regimens being used. The FIT group sees the need for properly conducted prospective studies to evaluate the impact of adding emicizumab, and in the future, other non‐factor therapies, into the management of patients with inhibitors.

**Table 2:** Key conclusions and recommendations

Eradication of inhibitors is still a desirable goal and ITI is the only approach that currently offers this potential.
Patients with inhibitors should be offered at least one attempt at ITI.
Although inhibitor eradication is still a laudable goal, for those patients who for various reasons must delay or are unable to undertake ITI, emicizumab alone is now an option.
The likelihood of successful ITI is mainly on the basis of historical pre‐ITI peak titre and peak titre during ITI. ITI dose/regimen may be chosen according to patients’ risk group.
Monitoring should be done frequently (suggest monthly), and ITI dose/frequency can be adjusted depending on how the patient is doing (based on changes in inhibitor titre and bleeding phenotype).
For patients who are not appearing to be successful with ITI, adjustments to the ITI regimen can be undertaken. This includes switching FVIII products or intensifying the regimen. With the availability of emicizumab, the FIT group would in general not be supportive of adding immunosuppressive therapy.
In the future, patients are likely to undertake fewer courses of ITI making the initial course so much more important and making decisions regarding what FVIII product to use and what ITI regimen to use even more important.
Emicizumab can almost certainly be used concomitantly with FVIII during ITI to prevent bleeds. This may impact on the decision of what ITI regimen to use.
Many questions remain as to what to do with patients after successful ITI; can tolerance to FVIII be maintained without ongoing exposure to FVIII?

## DISCLOSURES

The FIT group was supported by an unrestricted grant from Grifols, S.A., Barcelona, Spain. All authors participated in the planning conferences leading to this manuscript and received partial or full reimbursement for expenses but otherwise did not receive any honoraria for their participation in any FIT group activities. All authors critically revised and approved the final article. MC has received honoraria for participation in advisory boards, speaker fees and research support from Baxalta/Shire/Takeda, Bayer, Biogen/Bioverativ, Biotest, CSL Behring, Grifols, Novo Nordisk, Octapharma, Pfizer and Roche. CE‐E has acted as a consultant and received speaker's fees and/or research funding from the following companies: Alnylam, Baxalta/Shire/Takeda, Bayer, Biotest, CSL Behring, Grifols, Kedrion, Novo Nordisk, Octapharma, Roche and Sobi. ES has received reimbursement for attending symposia/congresses and/or honoraria for speaking or for consulting from Baxalta/Shire/Takeda, Bayer, Bioverativ, CSL Behring, Grifols, Kedrion, Novo Nordisk, Octapharma, Pfizer, Roche and Sobi. JO has received reimbursement for attending symposia/congresses and/or honoraria for speaking or consulting and/or funds for research from Bayer, Biotest, Chugai, CSL Behring, Grifols, Novo Nordisk, Octapharma, Pfizer, Roche, Shire and Sobi. RL has received reimbursement for attending conferences from Bayer, CSL Behring, Octapharma, Shire and Sobi; consultancy payments from Novo Nordisk, Octapharma, Roche and Shire; speaker's fees from Bayer and Octapharma. BN has received research funding from Biogen and is a consultant for Grifols. AB has received reimbursement for attending symposia/congresses and/or speaker fees from Grifols, Kedrion, Novo Nordisk, Octapharma, Pfizer, Roche, Shire and Sobi/Biogen and funds for research from Novo Nordisk and Octapharma. SH has received speaker fees from Baxter, Grifols, Novo Nordisk and Pfizer; honorarium for advisory boards from Grifols and Shire and reimbursement for attending conferences from Baxter, Bayer, Grifols, Novo Nordisk and Pfizer. GY has received reimbursement as a consultant for Alnylam, Bayer, Bioverativ, CSL Behring, Genentech, Grifols, Kedrion, Novo Nordisk, Roche and Shire.

## References

[1] Gouw SC , van der Bom JG , Auerswald G , et al. Recombinant versus plasma‐derived factor VIII products and the development of inhibitors in previously untreated patients with severe hemophilia A: the CANAL cohort study. Blood. 2007;109:4693‐4697. 17218379 10.1182/blood-2006-11-056317

[2] Mancuso EM , Cannavó A . Immune tolerance induction in hemophilia. Haemophilia. 2015;5:331‐335.

[3] Gomez K , Klamroth R , Mahlangu J , Mancuso ME , Mingot ME , Ozelo MC . Key issues in inhibitor management in patients with haemophilia. Blood Transfus. 2014;12(Suppl 1):s319‐s329. 24333092 10.2450/2013.0246-12 PMC3934290

[4] Rota M , Cortesi PA , Steinitz‐Trost KN , Reininger AJ , Gringeri A , Mantovani LG . Meta‐analysis on incidence of inhibitors in patients with haemophilia A treated with recombinant factor VIII products. Blood Coagul Fibrinolysis. 2017;28:627‐637. 28678027 10.1097/MBC.0000000000000647

[5] Peyvandi F , Mannucci PM , Garagiola I , et al. A randomized trial of factor VIII and neutralizing antibodies in hemophilia A. N Engl J Med. 2016;374:2054‐2064. 27223147 10.1056/NEJMoa1516437

[6] Walsh CE , Soucie JM , Miller CH , United States Hemophilia Treatment Center Network . Impact of inhibitors on hemophilia A mortality in the United States. Am J Hematol. 2015;90:400‐405. 25616111 10.1002/ajh.23957

[7] Eckhardt CL , Loomans JI , van Velzen AS , et al. Inhibitor development and mortality in non‐severe hemophilia A. J Thromb Haemost. 2015;13:1217‐1225. 25912309 10.1111/jth.12990

[8] Kreuz W , Escuriola Ettingshausen C , Vdovin V , et al. First prospective report on immune tolerance in poor risk haemophilia A inhibitor patients with a single factor VIII/von Willebrand factor concentrate in an observational immune tolerance induction study. Haemophilia. 2016;22:87‐95. 26202305 10.1111/hae.12774

[9] Earnshaw SR , Graham CN , McDade CL , Spears JB , Kessler CM . Factor VIII alloantibody inhibitors: cost analysis of immune tolerance induction vs. prophylaxis and on‐demand with bypass treatment. Haemophilia. 2015;21:310‐319. 25688580 10.1111/hae.12621

[10] Brackmann HH , White GC , Berntorp E , Anderson T , Escuriola‐Ettinghausen C . Immune tolerance induction: what have we learned over time? Haemophilia. 2018;24(Suppl 3):3‐14. 10.1111/hae.13445 29543371

[11] Hay C , DiMichele D , on behalf of the International Immune Tolerance Study . The principal results of the International Immune Tolerance Study: a randomized dose comparison. Blood. 2012;119:1335‐1344. 22101900 10.1182/blood-2011-08-369132

[12] DiMichele DM , Hoots WK , Pipe SW , Rivard GE , Santagostino E . International workshop on immune tolerance induction: consensus recommendations. Haemophilia. 2007;13(Suppl 1):676‐22. 10.1111/j.1365-2516.2007.01497.x 17593277

[13] Oldenburg J , Mahlangu JN , Kim B , et al. Emicizumab prophylaxis in hemophilia A with inhibitors. N Engl J Med. 2017;377:809‐818. 28691557 10.1056/NEJMoa1703068

[14] Young G , Liesner R , Sidonio RF Jr , et al. Emicizumab prophylaxis provides flexible and effective bleed control in children with hemophilia A with inhibitors: results from the Haven 2 study. Blood. 2018;132(S1):632.

[15] Mahlangu J , Cerquiera M , Srivastava A . Emerging therapies for haemophilia − global perspective. Haemophilia. 2018;24(Suppl 6):15‐21. 10.1111/hae.13510 29878661

[16] Callaghan MU , Sidonio R , Pipe SW . Novel therapeutics for hemophilia and other bleeding disorders. Blood. 2018;132:23‐30. 29769259 10.1182/blood-2017-09-743385

[17] Peyvandi F , Cannavò A , Garagiola I , et al. Timing and severity of inhibitor development in recombinant versus plasma‐derived factor VIII concentrates: a SIPPET analysis. J Thromb Haemost. 2018;16:39‐43. 29080391 10.1111/jth.13888

[18] Kempton CL , Meeks SL . Toward optimal therapy for inhibitors in hemophilia. Blood. 2014;124:3365‐3372. 25428222 10.1182/blood-2014-05-577643

[19] Arruda VR , Doshi VS , Samelson‐Jones BJ . Emerging therapies for hemophilia: unanswered questions. F1000 Res. 2018;7:676‐12. 10.12688/f1000research.12491.1 PMC5931262 29770199

[20] Mariani G , Scheibel E , Nogao T , et al. Immunetolerance as treatment of alloantibodies to factor VIII in hemophilia. The International Registry of Immunetolerance Protocols. Semin Hematol. 1994;31(2 Suppl 4):62‐64. 7939781

[21] Lenk H , ITI Study Group . The German Registry of immune tolerance treatment−1999 update. Haematologica. 2000;85(10 Suppl):45‐47. 11187870

[22] DiMichele DM , Kroner B , North American Immune Tolerance Study Group . The North American Immune Tolerance Registry: practices, outcomes, outcome predictors. Thromb Haemost. 2002;87:52‐57. 11848456

[23] Jiménez‐Yuste V , Oldenburg J , Rangarajan S , Peiró‐Jordán R , Santagostino E . Long‐term outcome of haemophilia A patients after successful immune tolerance induction therapy using a single plasma‐derived FVIII/VWF product: the long‐term ITI study. Haemophilia. 2016;22:859‐865. 27329267 10.1111/hae.12986

[24] Caram C , de Souza RG , de Sousa JC , et al. The long‐term course of factor VIII inhibitors in patients with congenital haemophilia A without immune tolerance induction. Thromb Haemost. 2011;105:59‐65. 21057702 10.1160/TH10-04-0231

[25] Nakar C , Manco‐Johnson MJ , Lail A , et al. Prompt immune tolerance induction at inhibitor diagnosis regardless of titre may increase overall success in haemophilia A complicated by inhibitors: experience of two U.S. centres. Haemophilia. 2015;21:365‐373. 25581638 10.1111/hae.12608

[26] Liu CL , Lyle MJ , Shin SC , Miao CH . Strategies to target long‐lived plasma cells for treating hemophilia A inhibitors. Cell Immunol. 2016;301:65‐73. 26877251 10.1016/j.cellimm.2016.01.005 PMC4844017

[27] Oldenburg J , Jiménez‐Yuste V , Pieró‐Jordán R , Aledort LM , Santagostino E . Primary and rescue immune tolerance induction in children and adults: a multicentre international study with a VWF‐containing plasma‐derived FVIII concentrate. Haemophilia. 2014;20:83‐91. 24354480 10.1111/hae.12263

[28] Mannucci PM , Mancuso ME , Santagostino E . How to choose factor VIII to treat hemophilia. Blood. 2012;119:4108‐4114. 22411872 10.1182/blood-2012-01-394411

[29] Kreuz W , Ettingshausen CE . Inhibitors in patients with haemophilia A. Thromb Res. 2014;134(Suppl 1):S22‐S26. 24745722 10.1016/j.thromres.2013.10.016

[30] Rangarajan S , Jiménez‐Yuste V , Santagostino E . Adult haemophilia A patients with inhibitors: successful immune tolerance induction with a single FVIII/VWF product. Haemophilia. 2014;20:e399‐e443. 25333452 10.1111/hae.12521

[31] Collins P , Chalmers E , Alamelu J , et al. First‐line immune tolerance induction for children with severe haemophilia A: a protocol from the UK Haemophilia Centre Doctors' Organisation Inhibitor and Paediatric Working Parties. Haemophilia. 2017;23:654‐659. 28574205 10.1111/hae.13264

[32] Shapiro AD , Hedner U . Advances in bypassing agent therapy for hemophilia patients with inhibitors to close care gaps and improve outcomes. Ther Adv Drug Saf. 2011;2:213‐225. 25083214 10.1177/2042098611415566 PMC4110812

[33] Ullman AJ , Marsh N , Mihala G , Cooke M , Rickard CM . Complications of central venous access devices: a systematic review. Pediatrics. 2015;136:e1331. 26459655 10.1542/peds.2015-1507

[34] Collins PW , Fischer K , Morfini M , Blanchette VS , Björkman S , on behalf of International Prophylaxis Study Group (IPSG) Pharmacokinetics Expert Working group . Implications of coagulation factor VIII and IX pharmacokinetics in the prophylactic treatment of haemophilia. Haemophilia. 2011;17:2‐10. 20731726 10.1111/j.1365-2516.2010.02370.x

[35] Lenting PJ , Denis CV , Christophe OD . Emicizumab, a bispecific antibody recognizing coagulation factors IX and X: how does it actually compare to factor VIII? Blood. 2017;130:2463‐2468. 29042366 10.1182/blood-2017-08-801662

[36] Mahlangu J , Oldenburg J , Paz‐Priel I , et al. Emicizumab prophylaxis in patients who have hemophilia without inhibitors. N Engl J Med. 2018;379:811‐822. 30157389 10.1056/NEJMoa1803550

[37] Langer AL , Etra A , Aledort L . Evaluating the safety of emicizumab in patients with hemophilia A. Exp Opin Drug Saf. 2018;17:1233‐1237. 10.1080/14740338.2019.1551356 30462521

